# Zeolite for preventing periparturient hypocalcemia in dairy cows: mechanisms and application strategies

**DOI:** 10.3389/fvets.2025.1635245

**Published:** 2025-08-08

**Authors:** Xiu Su, Pengyu Huang, Yuanyin Guo, Jie Cao

**Affiliations:** Department of Clinical Veterinary Medicine, College of Veterinary Medicine, China Agricultural University, Beijing, China

**Keywords:** zeolite, transition dairy cows, hypocalcemia, calcium-phosphorus metabolism, rumen, immunomodulation

## Abstract

Preventing milk fever and subclinical hypocalcemia remains a critical challenge in high-producing dairy cows. This review focuses on the mechanisms of zeolite and the strategies for zeolite application as a novel approach for regulating calcium metabolism during the transition period. Zeolite is reported to reduce calcium absorption through ion exchange capacity in the digestive tract, pre-activating calcium homeostasis regulatory systems and consequently allowing cows to rapidly adapt to lactation calcium demands postpartum. In addition to directly affecting calcium and phosphorus utilization, zeolite may also optimize the periparturient mineral metabolism network by modulating the phosphate-FGF23-Klotho and serotonin-PTHrP-calcium axes. Additionally, the supplementation of zeolite stabilizes ruminal pH, improves volatile fatty acid composition, enhances fiber digestibility, and promotes dry matter intake, facilitating recovery from postpartum negative energy balance. Furthermore, zeolite exerts immunomodulatory effects, alleviating excessive inflammatory responses, oxidative stress, and periparturient systemic inflammation. However, type selection, dosage control, timing, and safety must be considered for zeolite application. Natural and synthetic zeolites exhibit differential efficacies owing to their unique structural characteristics and exchange capacity. The particle size and dosage of zeolite directly influence the degree of calcium-phosphorus metabolism regulation. Long-term usage of zeolites may present safety concerns, such as aluminum accumulation. Zeolite application strategies must be optimized based on the breed, physiological stage characteristics, and synergistic effects with other preventive measures to effectively manage periparturient hypocalcemia and promote overall dairy cow health and performance.

## 1 Introduction

Hypocalcemia, a common metabolic disorder in dairy cows during the transition period (7 days prepartum to 3 days postpartum), is characterized by serum calcium downregulation and recumbency (1). In addition to adversely affecting cow health and reducing milk production (2), hypocalcemia may lead to secondary diseases, such as mastitis, metritis, and abomasal displacement, contributing to economic losses to the global dairy industry (3). The major risk factors for hypocalcemia are parity, specific breeds, prepartum high-potassium diets, and magnesium deficiency (4, 5).

Postpartum hypocalcemia in dairy cows results from an imbalance between the sudden increase in calcium demand and the delayed response of calcium regulatory mechanisms. The daily requirement of calcium during the early lactation period is ~50 g (6), which is higher than the total blood calcium pool. To meet the increased demand, calcium is mobilized from the bone, and intestinal calcium absorption is upregulated. However, the efficiency of the calcium regulatory system in transition cows is dependent on factors, such as hypomagnesemia (7) and metabolic alkalosis (8) that lead to reduced parathyroid hormone (PTH) receptor sensitivity and delayed calcium regulatory responses. Thus, the increased calcium requirements of the early lactation stage cannot be met (9).

Currently, the preventive strategies for postpartum hypocalcemia include prepartum low-calcium diets (< 20 g/day) (10) and the addition of anionic salts to adjust dietary cation-anion difference recommended at −100–0 mEq/kg dry matter (DM) (11). However, these strategies are associated with several limitations. The major challenge in implementing low-calcium diets is decreasing the calcium content in feed ingredients, which is difficult to achieve in modern high-producing dairy cow formulations (9). Meanwhile, anionic salt supplementation is associated with various challenges, including poor palatability, decreased feed intake, complex monitoring requirements, and risks of excessive acidification (11, 12). Thus, efforts are ongoing to develop safe and effective alternatives.

Zeolites are a group of aluminosilicate minerals with unique ion exchange capabilities (13). In livestock production, zeolites are widely used as feed additives that adsorb harmful substances (such as toxins and heavy metal ions) in the gastrointestinal tract, improving intestinal health and promoting digestion and absorption (13–15). Additionally, zeolites reduce ammonia emissions, decreasing barn odors and improving the farming environment (13). The basic structure of zeolites comprises SiO_4_ and AlO_4_ tetrahedra connected through shared oxygen atoms, forming a three-dimensional framework with regular channels and cavity systems (16). Zeolites can be classified based on their origin as follows: natural zeolites (such as clinoptilolite), exhibit Si/Al ratios in the range of 4–7 and good chemical stability (17); synthetic zeolites (such as type A), industrially produced with Si/Al ratios close to one and exhibit enhanced ion exchange capacity (18). Zeolites can selectively bind several ions, including calcium and phosphorus ions, in the digestive tract, establishing a “functional low-calcium environment.” In contrast to traditional methods, the application of zeolites does not require the modification of diet formulation. Additionally, zeolites do not induce metabolic acidosis. Thus, the dietary supplementation of zeolites represents a novel approach to prevent postpartum hypocalcemia in dairy cows.

The preventive effects of zeolites on postpartum hypocalcemia are mediated through multiple mechanisms, including regulating calcium and phosphorus metabolism, optimizing the ruminal environment, modulating immune and inflammatory responses, and exerting multidimensional synergistic effects. However, zeolite application is associated with various challenges, such as differential efficacies of zeolite types, optimal dosage and particle size selection, potential aluminum accumulation risks, and safety issues with long-term usage. This review aimed to analyze the mechanisms of prepartum zeolite supplementation in preventing hypocalcemia in dairy cows with a focus on previous research findings, application strategies, potential risks, and solutions. Thus, the findings of this review may provide a reference for the application of zeolites in preventing postpartum hypocalcemia in dairy cows.

## 2 Zeolite and periparturient hypocalcemia in dairy cows

### 2.1 Zeolite and gastrointestinal calcium-phosphorus utilization

Research focusing on the mechanisms through which zeolites improve periparturient calcium metabolism has gradually evolved from phenomenological studies to mechanistic studies. The ion-exchange and adsorption properties of zeolites form the fundamental basis for their diverse applications in biological and environmental systems. Their crystalline framework comprises a highly ordered array of micropores and cavities, which selectively accommodate small molecules and ions, facilitating interactions with exchangeable cations embedded in the lattice structure (19). The efficiency and specificity of these interactions are governed by environmental conditions, including solution pH, ionic strength, and temperature (20). Early studies suggested that zeolites function through ion exchange mechanisms. Zeolites bind calcium ions in the digestive tract of dairy cows, temporarily decreasing calcium bioavailability, establishing a negative calcium balance state, and consequently activating the calcium homeostasis regulatory system.

Jørgensen et al. (21) reported an “overshooting” phenomenon after the oral administration of synthetic zeolite A. In this phenomenon, the blood calcium levels decrease slightly during treatment but increase above baseline levels after discontinuation. Thus, prepartum zeolite feeding “trains” the calcium regulatory system of dairy cows to rapidly respond to hypocalcemia risk after calving. Thilsing-Hansen and Jørgensen (22) revealed that zeolite A significantly increased the 1,25(OH)_2_D_3_ levels. Additionally, the plasma inorganic phosphorus levels were markedly downregulated in cows fed on zeolite A during the first week of postpartum. Thilsing et al. (23) performed *in vitro* studies and reported that the characteristics of zeolite A vary with pH conditions. Zeolite binds to calcium under ruminal pH conditions, partially releasing it in acidic environments. Subsequently, zeolite rebinds to calcium under small intestinal pH conditions. These findings are consistent with the theoretical model for hypocalcemia prevention. The calcium binding capacity of zeolite measured in trials was determined to be 7–19 mg/g, which is lower than the theoretical value (92 mg/g). This suggests the presence of additional regulatory mechanisms. Previous studies have reported that zeolite does not affect supernatant phosphorus concentrations under ruminal pH conditions. However, under small intestinal pH conditions, the phosphorus binding capacity of zeolite was significantly higher than the concurrent calcium binding capacity. This can explain the hypophosphatemia phenomenon observed in previous trials.

The effects of zeolite on phosphorus metabolic pathways were examined to understand the occurrence of hypophosphatemia. Thilsing et al. (24) reported that zeolite A slowly releases aluminum ions in the digestive tract, which can lead to the formation of insoluble aluminum phosphate complexes. Grabherr et al. (25) demonstrated that zeolite A begins decomposing and releasing aluminum ions in the rumen, significantly increasing soluble aluminum concentrations in the rumen and duodenum. Additionally, the serum phosphorus concentrations were negatively correlated with zeolite A intake. This suggests that zeolite can cause mild hypophosphatemia by reducing phosphorus bioavailability. Moderate restriction of phosphorus intake in periparturient dairy cows improves blood calcium homeostasis by enhancing (non-PTH dependent) bone resorption and regulating vitamin D activity (26). Therefore, the reduction of phosphorus utilization is an alternative mechanism through which zeolite improves calcium homeostasis.

### 2.2 Zeolite and the phosphate-FGF23-Klotho axis

Various studies have attempted to elucidate the mechanisms underlying the effect of zeolite on calcium balance through the regulation of phosphorus metabolism. Fibroblast growth factor 23 (FGF23), an endocrine factor secreted by osteocytes, in combination with Klotho protein is part of a signaling axis involved in regulating phosphorus and calcium metabolism (27). Klotho, which is a co-receptor for FGF23, enhances the binding of FGF23 to its receptor (FGFR), resulting in the activation of the downstream MAPK/ERK signaling pathway (a key pathway that regulates cell growth and function), upregulation of the phosphate-responsive gene Galnt3 (28), downregulation of the expression and activity of type II Na-dependent phosphate co-transporters (NaPi-2a and NaPi-2c) in renal proximal tubular epithelial cells, and suppression of phosphate reabsorption (29). Treatment with high concentrations of phosphate upregulated FGF23 levels in adult mouse osteocytes (30). FGF23 can also maintain precise calcium and phosphorus balance by inhibiting renal 1α-hydroxylase activity, suppressing 1,25(OH)_2_D_3_ synthesis, and decreasing intestinal calcium and phosphorus absorption efficiency (31).

**Figure 1:**
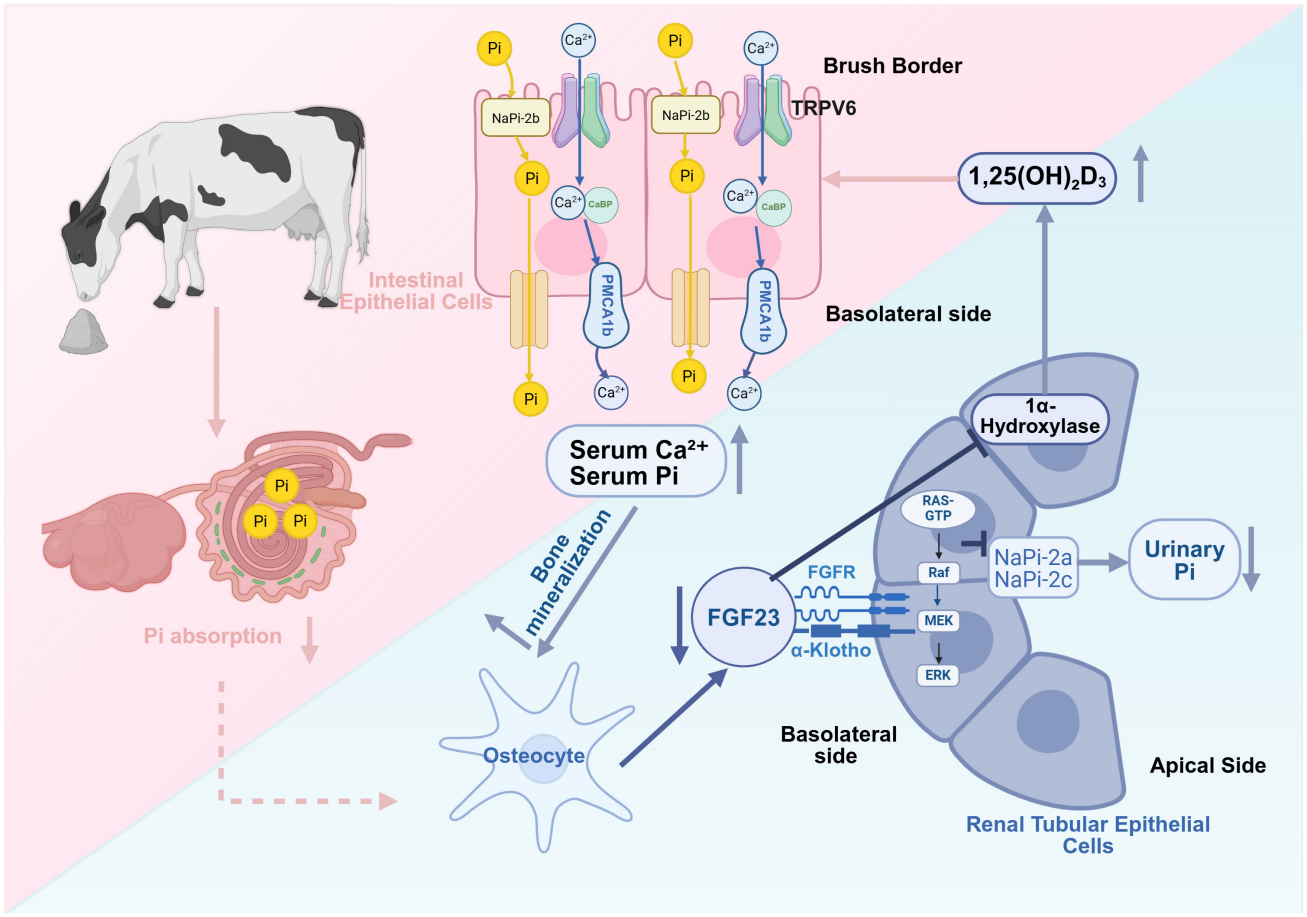
Zeolite may regulate calcium-phosphate homeostasis through the inhibition of the fibroblast growth factor 23 (FGF23)-Klotho signaling pathway. Zeolite decreases intestinal phosphorus (Pi) by binding to it. The downregulation of serum Pi suppresses osteocyte FGF23 production, weakening FGF23-Klotho complexes and mitogen-activated protein kinase/extracellular signal-regulated kinase (MAPK/ERK) signaling in renal tubular cells. This relieves the inhibition of renal sodium-dependent phosphate transporters 2a/2c (NaPi-2a/2c), intestinal sodium-dependent phosphate transporter 2b (NaPi-2b), and 1α-hydroxylase, enhancing renal Pi reabsorption and 1,25-dihydroxyvitamin D_3_ synthesis, promoting intestinal calcium and phosphorus absorption and bone calcium mobilization, and consequently improving periparturient calcium-phosphorus homeostasis (Created in https://BioRender.com).

Coordinated actions of FGF23 and its co-receptor Klotho enable rapid downregulation of circulating phosphate levels, thereby influencing calcium metabolism and modulating the synthesis of active vitamin D. Together, they constitute a complex feedback regulatory network essential for mineral homeostasis. Therefore, Frizzarini et al. (32) hypothesized that zeolite enhances bone resorption by downregulating FGF23 to support calcium demands in periparturient dairy cows. This hypothesis was based on experimental studies in which zeolite significantly decreased the blood and saliva phosphorus levels and increased the fecal water-soluble phosphate levels. Throughout the transition period, the average serum phosphorus levels in zeolite-fed cows were 52% lower than those in cows fed on anionic or control diets. The restriction of blood phosphorus may trigger a series of compensatory mechanisms through the FGF23-Klotho signaling pathway to maintain calcium levels during high lactation demands (28). Mouse and goat studies have revealed that zeolite decreased blood phosphorus levels without impairing bone mineralization and induced vitamin D fluctuation (33, 34). These findings indicate that under external mineral intervention conditions, pathways, such as FGF23-Klotho are used for new adaptive regulation of phosphorus and calcium to balance skeletal health and lactation requirements (Figure 1). However, this association remains speculative. Zeolite-induced dynamic changes in FGF23 or Klotho have not been systematically studied.

### 2.3 Zeolite and the serotonin-PTH-related protein (PTHrP)-calcium axis

The serotonin-PTHrP-calcium axis is a key calcium regulatory pathway. Serotonin is a neurotransmitter synthesized from L-tryptophan (Trp) via a two-step enzymatic process. Initially, L-tryptophan is hydroxylated by tryptophan hydroxylase 1 (TPH1) to form 5-hydroxytryptophan (5-HTP), which is subsequently decarboxylated by aromatic L-amino acid decarboxylase to yield serotonin (35). Beyond its well-established role in the central nervous system, serotonin also functions as a peripheral hormone involved in the regulation of diverse physiological processes across multiple organ systems. Previous studies have reported that 5-HT induces PTHrP expression and secretion through 5-HTR2B (36). Activated receptor mediates the activation of the downstream PLCβ3-ERK1/2 pathway, upregulating calcium transport proteins, including calcium release-activated calcium modulator 1 (ORAI1), and plasma membrane calcium pump (PMCA2) (36). The secreted PTHrP binds to PTH1R in bone tissue, stimulating bone resorption to release calcium and maintain blood calcium homeostasis (37). Studies employing mammary-specific PTHrP knockout mouse models have demonstrated that the absence of mammary-derived PTHrP results in reduced osteoclast numbers, decreased urinary levels of the bone resorption marker C-terminal telopeptide of type I collagen (CTX), and attenuated lactation-induced bone loss (38). Furthermore, in murine models of hypocalcemia induced by dietary calcium deficiency, inhibition of the calcium-sensing receptor (CaSR) signaling pathway alleviates its suppressive effect on PTHrP secretion, resulting in elevated circulating levels of PTHrP (39). Additionally, the mammary epithelial CaSR directly senses blood calcium fluctuations and modulates PTHrP production (37, 40). In mice with diet-induced hypocalcemia, the inhibition of the CaSR signaling pathway alleviates its suppressive effect on PTHrP secretion, promoting PTHrP production and release. The infusion of 5-HTP significantly upregulates mammary CaSR mRNA expression, suggesting that this pathway participates in calcium metabolism feedback regulation (41).

The role of 5-HT in bovine calcium metabolism has been well-established. Bovine mammary epithelial cells express complete serotonin synthesis pathway-related proteins and multiple serotonin receptor subtypes (42). The prepartum administration of 5-HTP in dairy cows enhances the levels of circulating 5-HT and attenuates postpartum calcium decline (43). Slater et al. (44) demonstrated that the combination of 5-HTP and anionic salt increased periparturient calcium stability. Previous studies have reported that the responses to 5-HTP intervention vary with breeds. For example, Holstein cows primarily enhance bone resorption, while Jersey cows develop transient prepartum hypocalcemia that facilitates postpartum calcium recovery (45), suggesting that the effects of zeolite may vary across different dairy cattle breeds.

**Figure 2:**
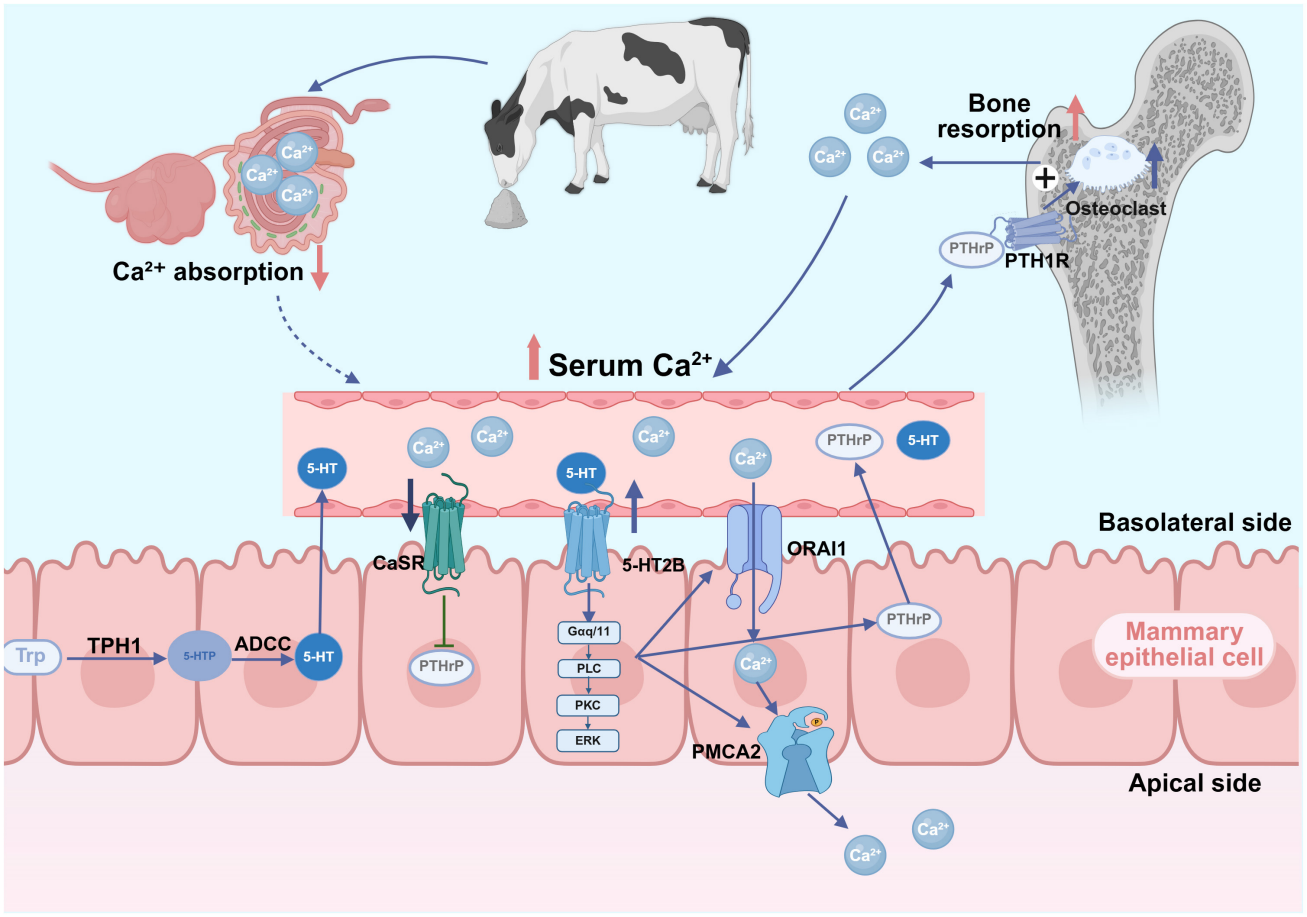
Proposed mechanism of the serotonin-parathyroid hormone-related protein (PTHrP)-calcium regulatory axis in zeolite-supplemented dairy cows. Dietary zeolite binds intestinal Ca^2+^, reducing its absorption and serum levels. The downregulation of calcium levels promotes the disinhibition of calcium-sensing receptor (CaSR) on the mammary epithelial cell surface, relieving its inhibitory effects on PTHrP synthesis. Hypocalcemia enhances serotonin (5-hydroxytryptamine, 5-HT) physiological activity. The binding of 5-HT to serotonin receptor 2B (5-HT2B) receptors activates signaling pathways that upregulate calcium transporters [calcium release-activated calcium channel protein 1 (ORAI1) and plasma membrane calcium ATPase 2 (PMCA2)], facilitating calcium transfer into milk, amplifying the hypocalcemic signal, and promoting PTHrP synthesis and secretion. Circulating PTHrP binds to bone parathyroid hormone receptor 1 (PTH1R), stimulating osteoclast-mediated bone resorption and calcium mobilization. The resulting upregulation of blood calcium reactivates mammary CaSR, inhibiting PTHrP production and completing a negative feedback loop that maintains calcium homeostasis (Created in https://BioRender.com).

Rodney et al. (46) revealed that anionic diet supplementation increased both prepartum 5-HT and C-terminal collagen crosslinked peptide (CTX-1) levels (a marker of bone resorption or breakdown), suggesting the serotonergic stimulation of bone resorption. Zeolite may indirectly (47) modulate calcium homeostasis through serotonergic pathways. The potential mechanism through which zeolite regulates calcium homeostasis via the serotonin-PTHrP pathway is shown in Figure 2. Frizzarini et al. (32) observed an increasing trend for serotonin concentrations in cows fed zeolite prepartum, although the difference did not reach statistical significance.

Based on the above findings, it may be hypothesized that zeolite-induced alterations in phosphorus absorption and FGF23 expression exert synergistic effects with serotonergic signaling to optimize bone calcium mobilization and blood calcium stability. However, quantitative data directly linking zeolite administration to changes in the serotonin-PTHrP axis are not available. Future studies must simultaneously monitor the zeolite supplementation-induced dynamic changes in 5-HT, PTHrP, and mineral parameters to elucidate these proposed mechanisms.

### 2.4 Zeolite and other minerals

Magnesium serves as an essential cofactor for various enzymes involved in bone formation and mineralization (48). Hypomagnesemia has been shown to suppress parathyroid hormone (PTH) secretion and induce resistance to PTH at target organs, thereby disrupting calcium and phosphate homeostasis (49). Watkins and Southern (47) reported that dietary sodium zeolite A supplementation does not alter blood magnesium concentrations in chicks but significantly downregulated tibial magnesium content. Schwaller et al. (33) and Abdelrahman et al. (34) demonstrated that synthetic zeolite A administration downregulated the blood magnesium levels in sheep. Frizzarini et al. (32) revealed that the serum magnesium concentrations in the zeolite-supplemented group from 14 days prepartum through day 1 postpartum were lower than those in the anionic diet and control groups in dairy cattle. Magnesium reached the lowest levels on day 1 postpartum. By day 6 postpartum, the zeolite group exhibited higher magnesium levels than other treatment groups although all values remained within the physiological range. These findings provide valuable insights into potential zeolite-magnesium-bone interactions that may contribute to calcium homeostasis. Limited direct evidence is available for a zeolite-magnesium-bone regulatory axis. warranting further investigation.

Additionally, some of the biological effects of zeolite may be attributable to the actions of silicon. Reffitt et al. (50) reported that orthosilicic acid stimulates type I collagen synthesis and promotes osteoblast differentiation in human osteoblast-like cells. Mladenović et al. (51) demonstrated that soluble silica inhibits osteoclast formation and bone resorption *in vitro*. Magnusson et al. (52) further confirmed that orthosilicic acid directly suppresses RANKL-induced osteoclastogenesis (the formation of bone-resorbing cells). These findings suggest that zeolite may influence bone remodeling and calcium homeostasis, at least in part, through the release of silicates in the gastrointestinal tract. However, direct experimental evidence supporting this hypothesis remains limited.

## 3 Effects of zeolite on ruminal function in dairy cows

### 3.1 Impact on ruminal pH and fermentation parameters

Ruminal fermentation and digestive function are critical components of ruminant metabolism. A stable pH is essential for maintaining microbial populations and nutrient utilization. Fluctuations in the ruminal environment are associated with mineral metabolism in dairy cattle (53). Dietary zeolite supplementation enhances the ruminal environment primarily through its cation exchange properties, enabling H^+^ ion adsorption and the subsequent pH increase (54, 55), a factor which, based on *in vitro* evidence (23), may itself influence the pH-dependent binding dynamics between zeolite and calcium ions within the rumen.

McCollum and Galyean (56) demonstrated that supplementing high-concentrate diets (85% concentrate) with 2.5% DM clinoptilolite significantly increased ruminal propionate levels and decreased the acetate:propionate ratio, suggesting improved energy utilization efficiency through the modification of volatile fatty acid (VFA) profiles. Consistently, Urías-Estrada et al. (57) reported that clinoptilolite administration upregulated the total VFA levels, downregulated acetate levels, and enhanced the propionate percentage in Holstein steers. However, Karatzia et al. (58) and Grabherr et al. (25) reported that zeolite supplementation decreased propionate and valerate levels and increased acetate levels in Holstein cows. These contrasting patterns have also been documented in small ruminants. Mahdavirad et al. (59) demonstrated that 2% natural zeolite supplementation maintained ruminal pH and increased the acetate:propionate ratio in lambs. El-Nile et al. (60) reported that nano-zeolite administration increased both total short-chain fatty acids and butyrate levels in goats. Additionally, the groups administered with nano-zeolite and natural zeolite exhibited decreased ruminal ammonia nitrogen (NH_3_-N) and increased acetate:propionate ratio when compared with the control group.

Several factors may account for these discrepancies, including variations in zeolite physicochemical properties (particle size and cation exchange capacity) that influence VFA adsorption dynamics. Additionally, forage-to-concentrate ratios significantly affect the efficacy of zeolite. Amanzougarene et al. (61) reported that pH modulation with high-concentrate diets (65:35) was more pronounced than that with low-concentrate formulations (35:65). Animal models (dairy vs. beef cattle), physiological status, and zeolite dosage may also contribute to discrepant results.

Most studies have reported the beneficial effects of zeolite in a specific dose range on ruminal fermentation. A meta-analysis by Khachlouf et al. (62) established that moderate zeolite doses (200–400 g/head/day) increase ruminal pH in lactating cows, enhance acetate proportion, and reduce propionate percentage. However, excessive supplementation of zeolite (>400 g/head/day) adversely affects fermentation parameters, which can be attributed to reduced dietary energy density (63) or fermentation imbalances (62).

Zeolite also modulates ruminal NH_3_-N metabolism. Sallam et al. (64) and Omarkozhauly et al. (65) revealed that 1%−2% zeolite supplementation reduced ruminal NH_3_-N concentrations and enhanced microbial protein synthesis in cattle and sheep, suggesting optimized nitrogen utilization.

Furthermore, natural zeolite (20 g/kg DM) or nano-zeolite (0.40 g/kg DM) significantly increased ruminal protozoa populations and cellulolytic enzyme activity in goats (60, 66), presumably by stabilizing ruminal pH and NH_3_-N concentrations and establishing favorable conditions for the growth of fibrolytic microbes. Omarkozhauly et al. (65) revealed that zeolite-chlorella complexes activated ruminal microbial ecosystems in dairy cows, enhancing both protozoa populations and amylolytic/cellulolytic enzyme activities. However, the stimulatory effects of zeolite on ruminal microbial proliferation have not been confirmed by all studies (61).

### 3.2 Effects on feed intake and digestibility

Alterations in the ruminal environment influence DM intake (DMI) and nutrient digestibility (67). Karatzia et al. (68) revealed improved body condition scores (BCS) in primiparous Holstein cows administered with clinoptilolite (200 g/day), which can attributed to enhanced DMI. Mahdavirad et al. (59) demonstrated that natural zeolite (2% DM) did not significantly affect DMI, average daily gain, or feed conversion ratio in lambs. This indicated that the feed intake in the natural zeolite-treated group was similar to that in the control group. Moderate zeolite supplementation (< 300 g/head/day) preserves physiological DMI patterns in lactating cows (62). However, zeolite supplementation at high doses (>400 g/head/day) significantly reduces DMI owing to decreased palatability, increases ruminal osmotic pressure, or downregulates plasma phosphorus levels (62, 63, 69).

Zeolites exert differential effects on digestibility. McCollum and Galyean (56) revealed that clinoptilolite (1.25% DM) significantly improved total dry matter digestibility (DMD) in beef cattle. The supplementation of clinoptilolite at high rates (2.5 and 5% DM) decreased DMD. Câmara et al. (63) reported that zeolite dose-dependently decreased total digestible nutrient digestibility, indicating dilution of dietary energy density by nutritionally inert zeolite material.

Ruminal zeolite enhances both fiber (63, 65) and starch digestion (56, 57, 65), mitigating the acidosis risk associated with rapid fermentation. Sallam et al. (64) demonstrated that supplementing 20 g clinoptilolite in urea-containing diets improved crude protein digestibility and feed conversion and decreased ruminal NH_3_-N concentration in lambs. Tables 1, 2 summarize the effects of different types and dosages of zeolite on ruminal function in various ruminant species. These inconsistent findings indicate that zeolite use should be adapted to specific animal and diet conditions, though further research is warranted.

**Table 1:** Effects of dietary zeolite supplementation on rumen function in cattle.

**Type of zeolite**	**Dosage**	**Animal species**	**Main effects**	**References**
Clinoptilolite	1.25, 2.5, 5% DM	Beef cattle (Zebu × British crossbred steers)	1.25% increased total DM digestibility; 2.5% increased propionate and decreased acetate/propionate (A/P) ratio; 2.5% and 5% decreased ruminal pH and NH_3_-N	McCollum and Galyean (56)
Zeolite A	10 and 20 g/kg DM	Dairy cows (Holstein)	No significant effect on ruminal pH or total SCFA concentration; increased acetate molar proportion, decreased propionate and valerate; increased A/P ratio; reduced DM and OM digestibility	Grabherr et al. (25)
Clinoptilolite	200 g/day	Dairy cows (Holstein)	Increased ruminal pH and acetate; decreased propionate and valerate molar proportions	Karatzia et al. (58)
Clinoptilolite	1.4% of TMR diet	Dairy cows (Holstein)	Tended to increase ruminal pH (*P* = 0.11); tended to decrease total VFA concentration (*P* = 0.14)	Dschaak et al. (55)
Clinoptilolite	0, 0.75, 1.5, 2.25, 3.0% DM	Beef cattle (Crossbred)	Linearly increased DM and NDF intake; linearly decreased total digestible nutrient digestibility with increasing dosage	Câmara et al. (63)
Clinoptilolite	2.5% DM (30 and 400 μm)	Beef cattle	No reduction in ruminal NH_3_-N; 30 μm particle size improved OM digestibility	Klaeui et al. (109)
Natural zeolite-chlorella top dressing	1% DM	Dairy cows (Simmental)	Slightly increased ruminal pH; increased VFA production, acetate molar proportion, and protozoa count	Omarkozhauly et al. (65)

This table summarizes findings from studies on both beef and dairy cattle, detailing the effects on ruminal pH, volatile fatty acid (VFA) profiles, and nutrient digestibility. SCFA, short-chain fatty acids; OM, organic matter; DM, dry matter; A/P ratio, acetate-to-propionate ratio; NH_3_-N, ammonia nitrogen.; VFA, volatile fatty acids.

**Table 2:** Effects of dietary zeolite supplementation on rumen function in small ruminants and *in vitro* models.

**Type of zeolite**	**Dosage**	**Animal species**	**Main effects**	**References**
Natural and nano zeolite	Natural: 20 g/kg DM; Nano: 0.4 g/kg DM	Goats (Barki)	Both increased ruminal pH and reduced NH_3_-N; nano zeolite increased total SCFA and butyrate; no significant effect on nutrient digestibility; both reduced methane production *in vitro*	El-Nile et al. (60)
Clinoptilolite	2% of base diet	Sheep (Arabi lambs)	Increased ruminal pH and acetate proportion; increased A/P ratio	Mahdavirad et al. (59)
Clinoptilolite	10 mg/g of total substrate	*In vitro*	Significantly increased pH in high-concentrate diets	Amanzougarene and Fondevila (61)
Clinoptilolite	20 g/day	Sheep (Barki ram lambs)	Increased DM intake and crude protein digestibility	Sallam et al. (64)
Natural and nano zeolite	Natural: 20 g/kg DM; Nano: 0.4 g/kg DM	Dairy goats (Damascus)	Both increased ruminal pH and reduced NH_3_-N; nano form increased total SCFAs and butyrate; neither affected DM intake	El-Nile et al. (66)

This table compiles findings from studies on goats and sheep, as well as from in vitro experiments, focusing on zeolite's impact on rumen fermentation parameters SCFA, short-chain fatty acids; DM, dry matter; A/P ratio, acetate-to-propionate ratio; NH_3_-N, ammonia nitrogen; VFA, volatile fatty acids.

**Figure 3:**
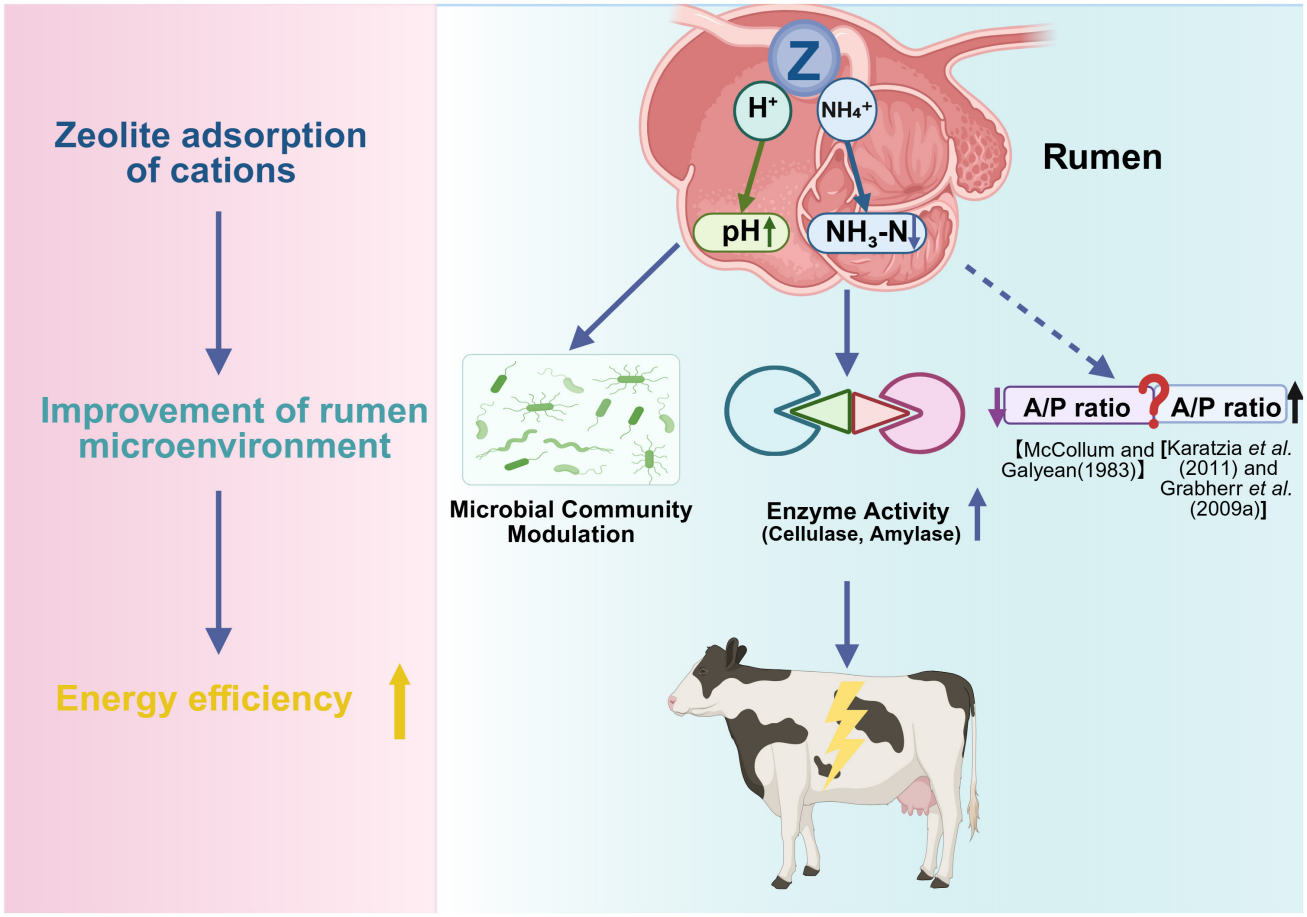
Proposed mechanism of zeolite in modulating rumen environment and fermentation. Zeolite exhibits cation exchange capacity to adsorb H^+^ and NH_4_^+^ ions, contributing to the stabilization of rumen pH and the downregulation of ruminal ammonia-N (NH_3_-N). These changes can optimize the rumen microbial ecosystem, promote protozoa growth and microbial protein synthesis, and enhance the activities of digestive enzymes, such as cellulase and amylase. Consequently, zeolite may alter the volatile fatty acid (VFA) profile and improve energy utilization efficiency. However, findings have been inconsistent on the effect of zeolite on the acetate/propionate (A/P) ratio (Created in https://BioRender.com).

As shown in Figure 3, appropriate zeolite supplementation (1%−2% DM or 200–400 g/head/day) enhances ruminal fermentation by stabilizing pH, reducing NH_3_-N concentrations, and promoting protozoa proliferation. These effects enhance microbial activity and dietary energy utilization, indirectly reducing postpartum hypocalcemia risk and other transition disorders (14, 70). However, inconsistent findings have been reported on the effect of zeolite on ruminal VFA profiles, especially acetate:propionate ratios, indicating the need for further studies.

## 4 Regulatory effects of zeolite on immune function and inflammatory responses

A critical interplay between the immune system and bone metabolism—a field known as osteoimmunology—characterizes the periparturient period. Systemic inflammation, common during this time, can exacerbate bone loss, as pro-inflammatory cytokines are potent stimulators of osteoclast-mediated bone resorption (38). This osteoimmune link provides a crucial context for zeolite's role beyond direct mineral binding. By mitigating periparturient inflammation and downregulating these cytokines, zeolite may offer an indirect yet significant mechanism for preserving bone integrity, complementing its primary effects on calcium and phosphorus metabolism.

### 4.1 Modulation of immune function

Zeolite enhances specific immune cell populations and activity through multiple pathways. Ivkovic et al. (71) demonstrated that tribomechanically activated zeolite clinoptilolite (TMAZ, a type of zeolite processed by intense mechanical grinding to increase its surface activity) stimulates lymphocyte proliferation and enhances the activities of CD4^+^ helper T cells and CD19^+^ B cells. Micronized clinoptilolite activates macrophage phagocytosis and bactericidal functions, promotes peritoneal macrophage infiltration, and increases reactive oxygen species (ROS) production (72). Valpotić et al. (73) revealed that clinoptilolite improves neutrophil phagocytic capacity and bactericidal activity in weaned piglets.

The supplementation of clinoptilolite to colostrum or regular milk can upregulate serum immunoglobulin (IgG) concentrations (74, 75). The serum γ-globulin, β-globulin, and total protein levels in groups fed on colostrum containing 0.5% clinoptilolite powder were higher than those in 2% clinoptilolite-fed and control groups (75). The supplementation of colostrum and regular milk with clinoptilolite at a dose of 0.5–1.0 g/kg body weight/day decreased the incidence and severity of diarrhea in newborn Holstein calves. However, the supplementation of clinoptilolite at doses higher than 0.5–1.0 g/kg body weight/day slightly inhibited IgG absorption, adversely affecting passive immunity and diarrhea outcomes (74). Karatzia et al. (54) demonstrated that prepartum clinoptilolite supplementation in combination with *Escherichia coli* vaccination significantly increased *E. coli*-specific antibody titers in maternal serum and upregulated antibody levels in the colostrum and calf blood. The effects were potentiated when zeolite was used in combination with selenium. However, synthetic zeolite A supplementation did not improve colostrum quality (76).

### 4.2 Effects of zeolites on inflammatory response

Various human and animal studies have demonstrated that zeolite attenuates inflammatory responses by modulating inflammatory signaling pathways. In rat primary hepatocyte culture models, clinoptilolite treatment significantly downregulated the adriamycin-induced upregulation of interleukin (IL)-1β, tumor necrosis factor (TNF)-α, and nuclear factor (NF)-κB expression levels. This suggests that clinoptilolite exerts anti-inflammatory effects by inhibiting the NF-κB pathway (77). Abu-Elfotuh et al. (78) used a manganese-induced Parkinson's disease rat model and demonstrated that micronized clinoptilolite combined with punicalagin significantly downregulated the TLR4/NF-κB/NLRP3 inflammatory pathway, alleviating neuroinflammation.

The anti-inflammatory effects of zeolite exhibited dose dependency in poultry animals. In particular, moderate doses (2% DM) downregulated acute phase proteins (tumor growth factor and C-reactive protein) and maintained immune homeostasis, while high doses (3% DM) upregulated pro-inflammatory cytokines (TNF-α, interferon (IFN)-γ, and IL-2), exacerbating inflammation (79). Human and *in vitro* studies have reported that clinoptilolite supplementation increases the levels of the anti-inflammatory cytokine IL-10 (80, 81).

In dairy cattle, periparturient clinoptilolite supplementation downregulated some inflammation-related genes (such as those encoding S100 family, CXCR1, and IFNG), decreased mastitis infection risk (82), and suppressed the levels of the acute phase proteins serum amyloid A and haptoglobin (83). Maity et al. (70) reported that clinoptilolite feeding downregulated complement components and coagulation factors involved in cascade reactions in postpartum bovine serum samples, mitigating inflammation and tissue damage.

Zeolite is also effective in mitigating oxidative stress. The unique physicochemical properties of zeolite, including large surface area, porous structure, and cation exchange capacity, enable toxin adsorption and free radical neutralization, suppressing ROS generation (72, 84, 85) and downregulating lipid peroxidation products, such as malondialdehyde (MDA) (86, 87). Additionally, zeolite enhances the activities of the antioxidant enzyme system, indirectly enhancing the elimination of ROS (88, 89). Ipek et al. reported that zeolite feeding downregulates plasma peroxide (LOOH) concentrations in dairy cows. However, the effect of zeolite on total antioxidant capacity (TAC) was limited in healthy cows. In meat rabbits, 1% clinoptilolite downregulated hepatic MDA levels without significantly affecting TAC (87). These findings suggest that the antioxidant effects of zeolite may be pronounced under oxidative stress conditions.

### 4.3 Potential mechanisms of immune and inflammation regulation

**Figure 4:**
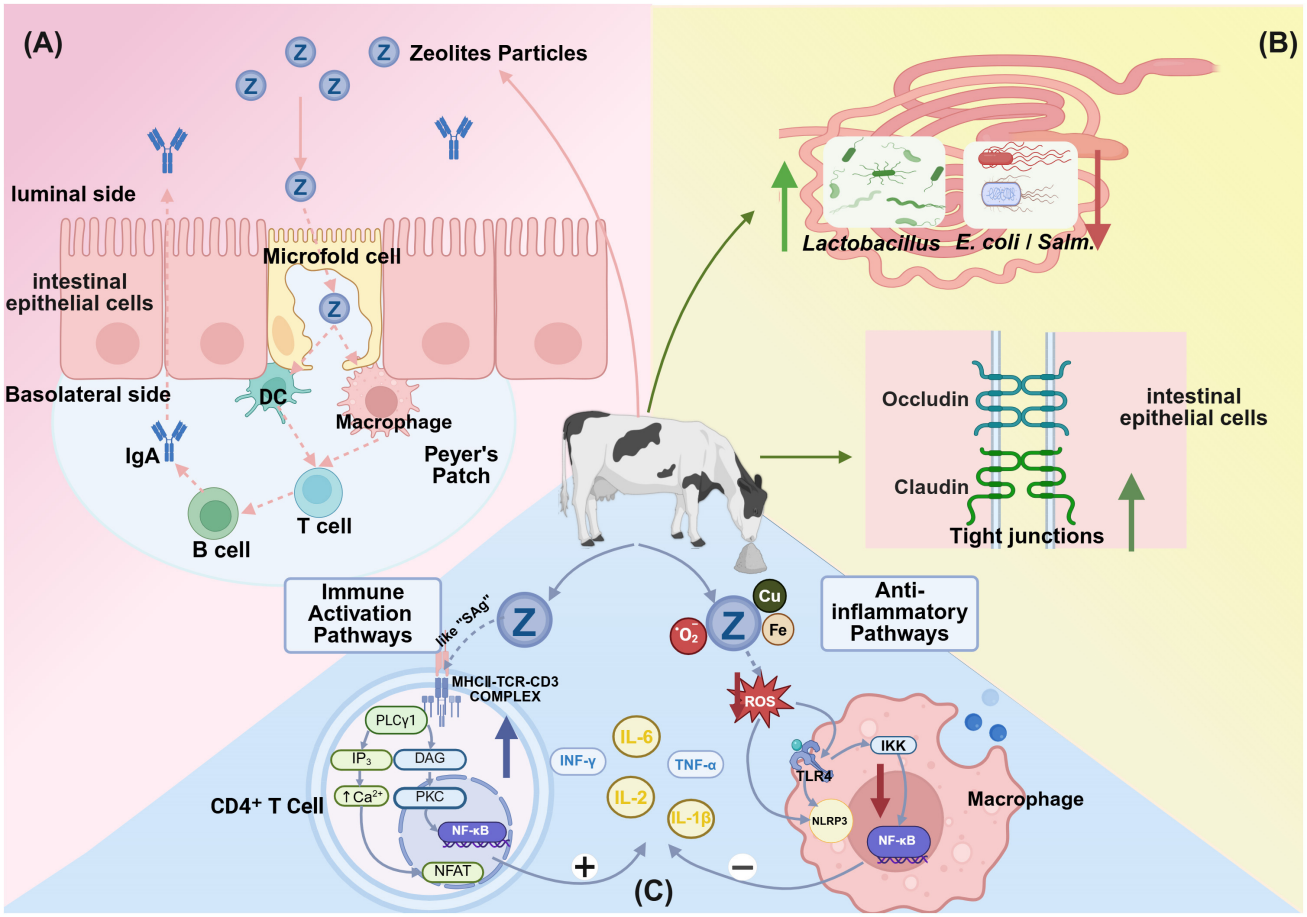
Proposed mechanisms through which dietary zeolite modulates immune responses and inflammation. **(A)** Zeolite particles are transcytosed via microfold (M) cells into Peyer's patches, where they are processed by antigen-presenting cells, such as dendritic cells and macrophages. This leads to B cell activation and immunoglobulin A (IgA) secretion, which are key elements of the intestinal mucosal immune response. *Adapted from Pavelić et al*. (91), with modifications. **(B)** Zeolite modulates the gut microbiota and barrier function by promoting the growth of beneficial bacteria (e.g., *Lactobacillus*) and suppressing the growth of pathogens (e.g., *E. coli* and *Salmonella*) and upregulating the expression of tight junction proteins (occludin and claudin) to maintain epithelial integrity. **(C)** Zeolite exerts bidirectional immunomodulatory and antioxidative effects. Zeolite may function as a superantigen to non-specifically cross-link the major histocompatibility complex Class II (MHC)-II-T cell receptor (TCR) complex in CD4^+^ T cells, activating the nuclear factor kappa B (NF-κB), nuclear factor of activated T cells (NFAT), and calcium signaling pathways and promoting the release of cytokines, such as interleukin-2 (IL-2), interferon gamma (IFN-γ), tumor necrosis factor alpha (TNF-α). Additionally, zeolite reduces intracellular reactive oxygen species (ROS) by adsorbing bacterial toxins and metal ions (e.g., Cu^2+^, Fe^2+^), alleviating oxidative stress and suppressing inflammatory signaling. This includes the downregulation of the toll-like receptor 4 (TLR4)-NF-κB pathway and the inhibition of ROS-dependent NOD-like receptor family pyrin domain containing 3 (NLRP3) inflammasome activation, leading to the downregulation of interleukin-1 beta (IL-1β), interleukin-6 (IL-6), and TNF-α (Created in https://BioRender.com).

The specific mechanisms through which zeolite modulates immune function and inflammation have not been completely elucidated but may involve multiple direct and indirect pathways (Figure 4).

The primary mechanism of zeolite may involve the direct adsorption of toxins, heavy metals, and harmful gases (such as ammonia) through its porous structure, mitigating toxicity and inflammatory stimulation (13). Additionally, zeolite may indirectly influence systemic immune and inflammatory status by regulating gut microbiota and intestinal barrier function. Wu et al. (84) revealed that dietary supplementation with 2% natural or modified clinoptilolite significantly reduced intestinal *E. coli* and *Salmonella* counts and increased the *Lactobacillus* abundance and intestinal villus height in broiler chickens. Additionally, zeolite can optimize gut microecology and enhance intestinal mucosal barrier function through the regulation of tight junction proteins (such as zonulin), resulting in the suppression of pathogen translocation and inflammatory responses (80, 90). Kraljević Pavelić et al. (91) postulated that zeolite stimulates intestinal microfold (M) cells, facilitating its entry into gut-associated lymphoid tissues, such as Peyer's patches, where it locally activates lymphocytes, dendritic cells, and macrophages, triggering mucosal and systemic immune responses. In contrast to most intestinal epithelial cells, M cells have weaker or non-existent mucus layers and microvilli but exhibit highly specialized capacity for efficient uptake and transport of macromolecules or microbial particles from the intestinal lumen to Peyer's patches (92). This “zeolite-gut immunity” hypothesis can explain the oral zeolite administration-induced coordinated changes in cellular and humoral immunity. However, Nizet et al. (93) performed histological examinations of mouse intestines and livers and did not detect clinoptilolite particles that crossed the intestinal barrier or accumulated in the tissues, contradicting Pavelić's hypothesis. Therefore, further verification studies are needed to investigate the potential biochemical behavior of clinoptilolite in the gastrointestinal tract (such as solubility tests).

Transition metals, such as copper (Cu) and iron (Fe) are essential for physiological metabolism. In their free state, these transition metals can catalyze Fenton reactions, promoting ROS generation (94). Stanojević et al. (95) reported that clinoptilolite pretreatment significantly downregulated iron content in the whole brain and the O_2_•- levels in the cerebellum and hippocampus of a mouse radiation injury model. Fan et al. (96) revealed that clinoptilolite mitigated iron overload-induced oxidative damage and pathological changes in organs. Additionally, trace elements, such as zinc and copper are involved in immune and antioxidant enzyme activities (97, 98). Zeolite feeding is reported to alter the serum or hepatic Zn and Cu levels in dairy cows, potentially affecting the expression of antioxidant enzymes, such as Cu-Zn superoxide dismutase (SOD) (99).

Several hypotheses have been proposed for the molecular mechanisms underlying the immunomodulatory effects of zeolite but have not been validated. The primary chemical components of zeolite are aluminosilicates. Silicate compounds are reported to exhibit immunostimulatory activity (100, 101). Horse and dog studies have reported that synthetic zeolite A can release trace amounts of H4SiO4 and aluminum ions under acidic conditions (102, 103). Ueki et al. (100) performed *in vitro* experiments to demonstrate that silicate materials, such as asbestos fibers can induce human peripheral blood mononuclear cell (PBMC) proliferation, upregulate CD4+ T cell proportions, and induce sustained elevation of intracellular Ca^2+^ levels. The cytokine secretion profile of PBMCs after asbestos stimulation was similar to that after superantigen stimulation. Additionally, asbestos stimulation markedly upregulated IL-2, a process dependent on major histocompatibility (MHC)-II. Therefore, the authors proposed that zeolite silicates function as “superantigen-like” substances (materials that can non-specifically activate a large number of immune cells), directly cross-linking MHC-II molecules and specific T cell receptor (TCR) Vβ regions, triggering rapid T cell proliferation and cytokine storms, and playing crucial roles in disease pathogenesis (104).

Zeolite may influence immune and inflammatory responses by regulating classical inflammatory signaling pathways, such as the NF-κB pathway and the NLRP3 inflammasome (77, 78). These pathways are involved in the pathogenesis of various inflammatory diseases. The regulatory effects of zeolite on these pathways may represent the key mechanism underlying its broad-spectrum anti-inflammatory effects.

## 5 Factors influencing zeolite application and safety considerations

### 5.1 Impact of zeolite characteristics on application efficacy

Zeolites are potential feed additives for improving calcium homeostasis in dairy cattle. However, the physicochemical properties and biological effects of zeolites vary depending on the types as shown in Table 3. Natural clinoptilolite is relatively stable in an acidic gastrointestinal environment and does not significantly upregulate serum aluminum levels (23, 58). Synthetic zeolite A exhibits enhanced cation exchange capacity and can effectively bind dietary calcium and phosphorus at low-to-moderate doses to prevent hypocalcemia (70). However, high doses of synthetic zeolite A can decrease available ruminal calcium, disrupting microbial activity and digestive function (23).

**Table 3:** Assessment of the effects of different zeolite types on key physiological mechanisms in dairy cows.

**Mechanisms**	**Zeolite A**	**Clinoptilolite**	**References**
Gastrointestinal calcium binding	Strong	Medium	(21, 22, 122, 124–126)
Effects on hypophosphatemia	Strong	Low to medium	(24, 32, 58, 69, 76, 126)
Effects on hypomagnesemia	Medium	Low to medium	(24, 32, 76, 126, 127)
Rumen fermentation regulation	Weak	Strong	(25, 55, 58, 60–62, 108)
Effect on immune modulation	Medium	Strong	(32, 74, 82, 83)
Effects on aluminum accumulation	Strong	Low	(23–25, 58)
Effects on reproductive performance	Low to medium	Strong	(14, 62, 76, 120, 125, 128)

Modern processing technologies have expanded the application potential of zeolite. Vibration-activated and micronized clinoptilolite exerts beneficial effects on metabolism at low doses (100 g/day), which can be attributed to the enhanced activity resulting from processing (105, 106). Dolanc et al. (107) demonstrated that panaceo micro activated (PMA) zeolite effectively reduced various toxic metal concentrations when compared with standard mechanically activated clinoptilolite.

Beyond the intrinsic properties of the zeolite type, the dosage and particle size are also critical factors that dictate its effects. Zeolite supplemented in dairy cattle diet exerts dose-dependent effects. Moderate zeolite doses (< 300–400 g/head/day or 23 g/kg DM) effectively enhance milk production and prevent hypocalcemia without significantly affecting DMI (62, 69). However, zeolite markedly decreases DMI (from 10.1–10.9 to 7.3 kg/day) at high doses (e.g., >400–500 g/head/day or 43 g/kg DM), directly reducing lactating cow performance (62, 69). Previous studies indicate that supplementation with clinoptilolite at a dosage of 2.5% DM significantly reduces ketosis incidence and optimizes milk production efficiency. Meanwhile, supplementation with clinoptilolite at a dosage of 1.25% DM did not yield the expected results, suggesting an optimal activity range for zeolite application (108).

Particle size is another critical factor influencing the efficacy and safety of zeolite. Fine-particle zeolite (30 μm) affects organic matter digestibility more than coarse-particle zeolite (400 μm). Additionally, fine-particle zeolite effectively reduces ruminal ammonia concentration and urea nitrogen excretion, while coarse-particle zeolite exhibits enhanced performance in reducing fecal ammonia emissions (109, 110). Compared with conventional zeolites, nano-zeolites achieve similar or superior results at low doses (0.4 vs. 20 g/kg DM for traditional zeolites) owing to their enhanced surface area and cation exchange capacity (60, 66). Enhancing the ionic exchange activity through nanoscale modification may also increase the risk of interference with essential minerals, such as calcium and phosphorus at high doses.

### 5.2 Safety assessment

While optimizing for efficacy, the safety of zeolite application warrants careful consideration, with aluminum accumulation being Aluminum accumulation is the major safety concern associated with zeolite application. Systemic accumulation can result in aluminum crossing the blood-brain barrier, adversely affecting neural tissue, disrupting microtubule protein assembly, and promoting cognitive decline (111). Additionally, systemic aluminum accumulation inhibits bone mineralization, potentially inducing osteomalacia (112).

The stability of zeolite in the acidic gastrointestinal environment directly affects the release of aluminum. Karatzia et al. (58) reported that the ruminal and serum aluminum concentrations were not significantly different between the clinoptilolite-supplemented and control groups. This suggests that natural clinoptilolite offers enhanced safety regarding aluminum accumulation. Modified products, such as PMA zeolite and oxygenated zeolite (PMA-O2) exhibit enhanced aluminum detoxification efficacy in aluminum oxide-intoxicated rats, whereas synthetic zeolite A increases the plasma aluminum levels (113). This suggests that specially processed natural zeolites exhibit structural stability and selective adsorption capacity, potentially reducing aluminum accumulation risk.

Long-term zeolite administration safety evaluations have yielded favorable results. Katsoulos et al. (108, 114–116) revealed that continuous supplementation with clinoptilolite (1.25 or 2.5% DM) from 4 weeks prepartum through the next dry period did not result in adverse effects on serum trace element concentrations, liver function indicators, blood urea nitrogen, total protein, or fat-soluble vitamin levels in dairy cows, supporting its relative safety for long-term usage. In a 4-year long-term clinical trial involving patients with osteoporosis, supplementation with PMA-zeolite did not result in the accumulation of heavy metals such as lead (Pb), aluminum (Al), or nickel (Ni) in the bloodstream. On the contrary, the treatment demonstrated potential detoxifying effects. Notably, transient reductions in mineral levels, including calcium, sodium, and copper, were observed during the mid-phase of the intervention (117).

Potential indirect effects of long-term zeolite usage must be examined. Grünberg et al. (118) suggested that even without apparent liver dysfunction, hypophosphatemic conditions may indirectly affect hepatic function through the induction of anorexia and the exacerbation of negative energy balance. Additionally, Eisenberg et al. (119) examined the effects of dietary phosphorus deficiency on periparturient dairy cow leukocyte function. The authors reported that severe hypophosphatemia (< 0.5 mmol/L) may slightly impact granulocyte count and phagocytic activity. These findings suggest that the long-term use of phosphorus-reducing zeolite products adversely affects bovine immune function. These effects remain minimal at standard dosages but can amplify under specific conditions, such as under high production or stress conditions.

Moreover, zeolite exerts beneficial effects on reproductive parameters. Compared with those in the control groups, the BCS, blood glucose, and reproductive parameter levels were higher and the ketone levels were lower in primiparous dairy cows supplemented with clinoptilolite (68). Additionally, compared with the control group, the clinoptilolite-supplemented group exhibited decreased postpartum to first service interval (DFS; 115.1 vs. 124.2 days), enhanced ovarian cyclical activity resumption, and increased 305-day milk yield (8,325.5 vs. 8,050 kg) (120). Similarly, daily supplementation with 400 g of clinoptilolite during the periparturient period significantly enhanced the reproductive performance of Romanian Spotted cows, as indicated by a shorter DFS and a lower average number of inseminations required per conception (121). Furthermore, clinoptilolite supplementation decreased the uterine polymorphonuclear neutrophil (PMN) counts and subclinical endometritis prevalence, shortened open periods, and improved 150-day pregnancy rates (14, 122).

Regarding productive performance, Ilic et al. (123) demonstrated that 2% zeolite supplementation effectively enhanced milk yield and fat production. Meanwhile, Dschaak et al. (55), Khachlouf et al. (62), and Đuričić et al. (106) reported minimal or non-significant effects of zeolite on milk production and milk fat percentage. These discrepancies may be related to experimental conditions, zeolite type, dosage level, and dietary formulation. Several studies have reported that zeolite reduced the fat-protein ratio, postpartum beta-hydroxybutyrate (BHBA), and non-esterified fatty acid levels (106, 121, 122), suggesting that zeolite may improve energy status by alleviating negative energy balance, which may potentially benefit early lactation cows that are prone to developing ketosis.

In summary, the practical application of zeolite is complex. Its efficacy appears to depend on a combination of factors, including the material's specific properties (such as purity and cation exchange capacity) (16), as well as application parameters like dosage, particle size, and timing. As the literature does not yet support a single, definitive optimization strategy, tailoring these variables to specific production systems remains a key area for future investigation.

## 6 Conclusions

Zeolite prevents periparturient hypocalcemia in dairy cows through multiple mechanisms. Its primary role involves reducing the bioavailability of dietary calcium and phosphorus via ion exchange, which pre-activates the cow's endogenous homeostatic systems. This effect may be synergistically supported by other functions, including the stabilization of the ruminal environment, which improves digestive health and energy status, and its immunomodulatory properties that can alleviate periparturient inflammation and oxidative stress. The efficacy of this approach, however, is dependent on key factors such as zeolite type, dosage, and particle size, which must be carefully considered in practice.

Future research should focus on elucidating several key areas to refine the application of zeolite. First, the precise molecular mechanisms and direct evidence for the interaction between zeolite and the FGF23-Klotho and serotonin-PTHrP axes are not yet fully understood and warrant deeper exploration. From a practical standpoint, further studies are needed to optimize application strategies, including determining the ideal zeolite characteristics (e.g., purity, cation exchange capacity) for different production systems. Moreover, long-term biosafety requires continued investigation, with a particular focus on the potential for aluminum accumulation not only in the cow's tissues but also its possible transfer into milk, which is a critical food safety consideration. Furthermore, to fully assess the potential impact of zeolite on osteoporosis risk, future trials should include direct monitoring of postpartum bone turnover markers. Answering these questions will be crucial for the wider and more precise use of zeolite in the dairy industry.
